# Stuttering Severity Modulates Effects of Non-invasive Brain Stimulation in Adults Who Stutter

**DOI:** 10.3389/fnhum.2019.00411

**Published:** 2019-11-21

**Authors:** Emily O’Dell Garnett, Ho Ming Chow, Ai Leen Choo, Soo-Eun Chang

**Affiliations:** ^1^Department of Psychiatry, University of Michigan, Ann Arbor, MI, United States; ^2^Nemours/Alfred I. DuPont Hospital for Children, Wilmington, DE, United States; ^3^Department of Communication Sciences and Disorders, University of Delaware, Newark, DE, United States; ^4^Department of Communication Sciences and Disorders, Georgia State University, Atlanta, GA, United States

**Keywords:** speech, stuttering, fMRI, tDCS, neuromodulation, fluency, neuroimaging

## Abstract

Stuttering is a neurodevelopmental disorder that manifests as frequent disruptions in the flow of speech, affecting 1% of adults. Treatments are limited to behavioral interventions with variable success and high relapse rates, particularly in adults. However, even in severe cases, fluency can be temporarily induced during conditions in which the speaker synchronizes his speech with external rhythmic cues, such as when reading in unison (choral speech) or with a metronome. Non-invasive neuromodulation techniques such as transcranial direct current stimulation (tDCS) have shown promise in augmenting the effects of behavioral treatment during motor and speech/language rehabilitation, but only one study to date has examined behavioral modulatory effects of tDCS in the context of stuttering. Using high-definition (HD)-tDCS electrodes, which improves focality of stimulation relative to conventional tDCS, we investigated the effects of tDCS on speech fluency and brain activation in 14 adults who stutter (AWS). Either anodal or sham stimulation was delivered on separate days over left supplementary motor area (SMA). During stimulation, participants read aloud in sync with a metronome. Measures of speech fluency and brain activity functional magnetic resonance imaging (fMRI) were collected before and after stimulation. No significant differences in brain activity or speech fluency were found when comparing active and sham stimulation. However, stuttering severity significantly modulated the effect of stimulation: active stimulation attenuated the atypically strong association between stuttering severity and right thalamocortical network activity, especially in more severe speakers. These preliminary results warrant additional research into potential application of HD-tDCS to modulate speech motor networks to enhance fluency in stuttering.

## Introduction

Adults with persistent developmental stuttering report life-long struggles in speech communication, leading to detrimental effects on their social, emotional, and vocational well-being (Craig et al., 2009; Yaruss, 2010). Conventional behavioral speech therapy requires a considerable amount of effort (>100 h) and still over 70% of those that undergo therapy experience relapse of stuttering symptoms (Andrews et al., 1980; Craig and Hancock, 1995; Craig et al., 1996; Hancock et al., 1998). Success with electronic devices using delayed auditory feedback is variable and short-lived (Foundas et al., 2013), and pharmacological treatments have variable effects across individuals who stutter and are associated with unwanted side effects (Bothe et al., 2006; Maguire et al., 2010). Therefore, there is a critical need for developing therapeutic interventions that result in long-term enhancement of speech fluency in people who stutter (PWS). There is accumulating evidence of: (a) subtle neural differences in PWS; and (b) that the use of non-invasive brain stimulation improves motor and language performance in individuals with neurological speech and motor impairments and healthy individuals. The present study investigated the effects of brain stimulation (*via* high definition transcranial direct current stimulation; HD-tDCS) on neural activity and speech fluency in adults who stutter (AWS).

Corroborating predictions based on a neurobiologically plausible model of speech production [Gradient Order Directions into Velocities of Articulators (GODIVA); Bohland et al., 2010], accumulating empirical findings have suggested that the core deficit in stuttering may stem from disruption in the basal ganglia thalamocortical (BGTC) network, specifically in connections among the basal ganglia, supplementary motor area (SMA), and ventral premotor cortex (vPMC; Civier et al., 2013). The disruption in the BGTC network affects coordination of precisely timed movement initiation (SMA), activation of speech sounds (vPMC), and sequencing of sounds (putamen), all needed for speech fluency (Bohland et al., 2010; Civier et al., 2013). The SMA is critical in planning complex movement routines, including those that are required for fluent speech production, that are *internally timed and initiated*, rather than in response to external cues (Cunnington et al., 1996; Packman et al., 2007). PWS demonstrate difficulty in preparing and controlling precisely-timed complex movements including speech, poor performance on non-speech behavioral tasks that involve timing and rhythm and attenuated functional connectivity among BGTC regions (Packman and Onslow, 2002; Packman et al., 2007; Lu et al., 2009, 2010; Chang and Zhu, 2013; Wieland et al., 2015; Chang et al., 2016). Interestingly, most PWS exhibit temporarily increased fluency during *externally paced* conditions such as choral speech or metronome-paced speech (Park and Logan, 2015). They also show more “normalized” brain activation patterns during such conditions (i.e., similar to nonstuttering speakers; De Nil et al., 2003; Preibisch et al., 2003; Neumann et al., 2005; Giraud et al., 2008; Kell et al., 2009; Toyomura et al., 2011, 2015).

As a treatment, however, such techniques remain inadequate, as any improvements in speech fluency are short-lived (Kell et al., 2009) and the benefits often are outweighed by reduction in speech naturalness. To date, neuroscience-based treatments for stuttering have been limited. Techniques that exploit the principles of neuroplasticity, such as neuromodulation with tDCS, may offer new insights. This technique is safe, well-tolerated, non-invasive, inexpensive, and portable (Bikson et al., 2016) compared to other brain stimulation techniques such as transcranial magnetic stimulation (TMS). Additionally, tDCS readily lends itself to double-blinded, sham-controlled studies suitable for clinical trials (Woods et al., 2016). Conventional tDCS sponge electrodes are large (e.g., 35 cm^2^), resulting in diffuse current spread, but focality has recently been improved with high definition (HD) electrodes, which were used in the present research (Nitsche et al., 2007; Datta et al., 2008, 2009; Kuo et al., 2013). Stimulation *via* HD-tDCS can either increase or decrease brain activity using a weak constant electrical current (1–2 mA). Unlike TMS, which can induce neural firing, tDCS affects the resting membrane potential through the modulation of cellular-level changes, including sodium and calcium-dependent channels and NMDA receptor activity, thereby promoting long-term potentiation/depression (LTP/LTD) like effects (Stagg et al., 2009; Fritsch et al., 2010; Bolognini et al., 2011; Stagg and Nitsche, 2011).

Brain stimulation in conjunction with functional magnetic resonance imaging (fMRI) can enable researchers to examine any changes in brain activity in the targeted region and functionally connected regions as a result of the stimulation (Baudewig et al., 2001; Lang et al., 2005; Jang et al., 2009; Stagg et al., 2009; Keeser et al., 2011; Polanía et al., 2012; Pereira et al., 2013; Hampstead et al., 2014; Liew et al., 2014; Weber et al., 2014; Holland et al., 2016). HD-tDCS has been used in previous studies to provide functional improvement in language, motor skill learning, verbal fluency, speech reaction time, naming, numerical processing abilities, spelling, and learning an artificial grammar (Iyer et al., 2005; Sparing et al., 2008; Dockery et al., 2009; Reis et al., 2009; Baker et al., 2010 ; Fertonani et al., 2010; de Vries et al., 2010; Cattaneo et al., 2011; Fiori et al., 2011; Fridriksson et al., 2011; Madhavan and Shah, 2012; Richardson et al., 2014; Tsapkini et al., 2014; Vestito et al., 2014; Hartwigsen, 2015; Malyutina and den Ouden, 2015; Allman et al., 2016; Cappon et al., 2016; Hashemirad et al., 2016; Woods et al., 2016). Effects of stimulation are not always uniform across participants even in a single study, which suggests the existence of “good” vs. “poor” responders. Clinical measures of initial severity, baseline task performance, state and trait characteristics, and other individual differences often predict response to treatment (Berryhill and Jones, 2012; Tseng et al., 2012; Sarkar et al., 2014; Benwell et al., 2015; Learmonth et al., 2015; Hsu et al., 2016). Such effects may mask significant findings at an overall or group level, only to be revealed in more nuanced planned analyses.

Only two tDCS studies have been published to date relevant to the treatment of stuttering. A recent study (Chesters et al., 2018) paired traditional tDCS targeting left IFG with fluency-inducing speech (i.e., metronome-timed speech, choral speech) in a randomized, double-blind, controlled study. Thirty AWS received either sham or active tDCS for 20 min on five consecutive days. Speech fluency during reading and conversation was measured before, during, and at 1 and 6 weeks after treatment. The group who received active stimulation showed significant decrease in stuttering at 1 week after treatment, compared to the training-only (sham stimulation) group. This was maintained at 6 weeks, but only in the reading condition. These results provide initial support for using non-invasive brain stimulation to improve speech fluency in AWS when applied consecutively for several days. Yada et al. (2019) found that cathodal traditional tDCS applied to right Broca’s area decreased stuttering, although the study design involved multiple block of stimulation per day with only a short washout period between blocks. Nevertheless, the positive impact on fluency lends additional support for using non-invasive brain stimulation in stuttering treatment.

Similar to Chesters et al. (2018), we tested the potential augmentative effects of pairing tDCS with metronome-timed speech on speech fluency and brain activity in regions within and connected to the BGTC core timing network in AWS. Our study differed from Chesters et al. (2018) in several ways: (1) we targeted the left SMA compared to the left IFG that was targeted in their study; (2) we used HD electrodes, which improves focality of stimulation; (3) we stimulated at 1.5 mA compared to 1 mA in their study; (4) we used a within-subject rather than a between-subject design; (5) we collected fMRI before and after the tDCS sessions to examine any changes in brain function associated with brain stimulation; and (6) our study was preliminary in nature, only including one session each per subject of active and sham stimulation. We had two primary research questions. First, can speech-related brain activity in AWS be modulated by a single dose of anodal HD-tDCS targeting SMA? This is a crucial first step in developing an empirical basis for further study of the use of neuromodulation as an augmentative agent in stuttering therapy. We expected that compared to sham stimulation (i.e., training alone with the metronome-timed speech), anodal stimulation over left SMA would increase activity in the SMA, left vPMC/IFG, basal ganglia regions. Second, does stuttering severity modulate the effects of stimulation on brain activity? We hypothesized that stimulation would help reduce strong correlations between hyperactive brain areas and stuttering while enhancing activity in brain areas negatively correlated with stuttering severity.

## Materials and Methods

### Participants

We recruited 19 AWS to participate in this study. Of those 19, data from five were discarded from the final analyses due to: falling asleep during the MRI (*n* = 1), attrition related to time requirements of the study (*n* = 1), different MRI protocol used (*n* = 2), and incidental finding during MRI (*n* = 1). Thus, the final group included 14 AWS (three females) with a mean age of 22.6 years (range 18–46). All participants were monolingual, native English speakers with no history of psychiatric, developmental, cognitive, or speech and language disorders other than stuttering. All participants reported normal hearing and tested within normal limits on expressive and receptive language tests (Peabody Picture Vocabulary Test 4th edition; Expressive Vocabulary Test 2nd edition). Severity scores from the SSI-4 ranged from very mild (10) to very severe (41), while %SLD ranged from 2% to 32%. All participants reported receiving stuttering treatment, with duration of treatment ranging from a few days to a few years, primarily during their school years, but none received therapy within 1 year of study participation. The protocol was approved by the Institutional Review Boards of the University of Michigan Medical School (IRBMED). All subjects gave written informed consent in accordance with the Declaration of Helsinki.

### Study Design

**Figure 1:**
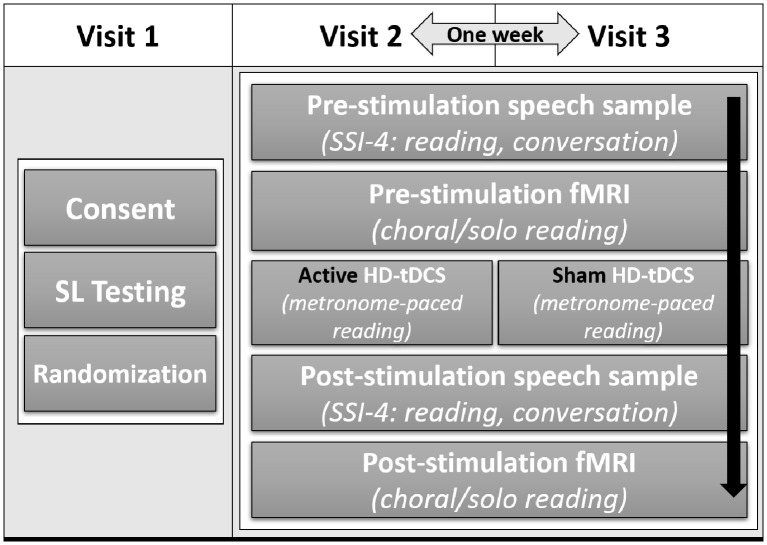
Study design showing group assignment, timeline, and task order for each visit. After consent and speech and language testing, participants were randomly assigned to receive active stimulation on visit 2 and sham stimulation on visit 3, or the reverse. All participants received active and sham stimulation on separate visits. Aside from the type of stimulation, visits 2 and 3 consisted of the same tasks in the same order for all participants.

This study employed a within-subjects, counterbalanced, sham-controlled, double-blind design over three visits (Figure 1). Visit 1 included obtaining informed consent, administering speech and language tests, and practicing the experimental tasks for the subsequent visits. The protocol was identical for visits 2 and 3, which were exactly 1 week apart, only the stimulation condition changed. This design allowed each subject to be his or her own control. Participants were randomized into one of two groups: one group received active stimulation on visit 2 and sham stimulation visit 3, with the order reversed in the other group (see “HD-tDCS procedure” section below for details on active and sham stimulation). fMRI data were collected before and after the tDCS session to assess effects of stimulation on brain activity. Speech samples were also collected before and after stimulation to assess effects of stimulation on speech fluency. Stuttering severity was assessed off-line by a certified SLP using the Stuttering Severity Instrument [(SSI-4); Riley and Bakker, 2009] who was blind to visit number and before/after stimulation.

### HD-tDCS Procedure

**Figure 2:**
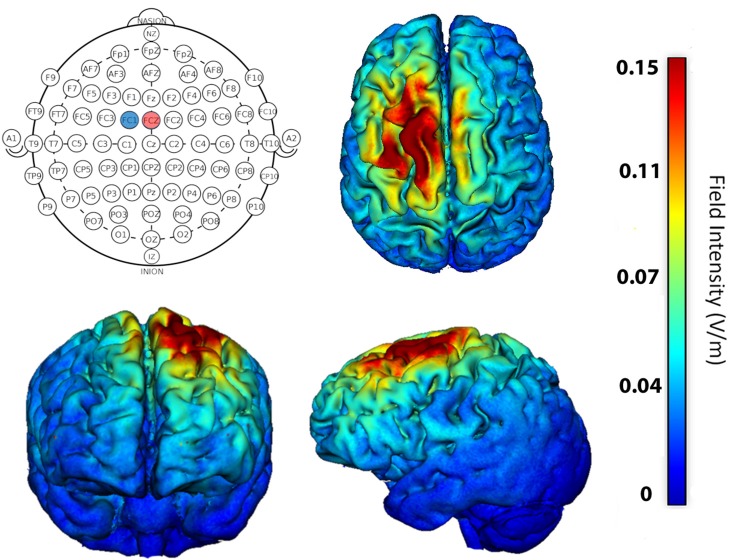
Stimulation location was determined using software that models current flow in the brain. For maximum focality stimulation of left supplementary motor area (SMA), the anode (+1.5 mA) was placed at FCZ (red circle) and cathode (−1.5 mA) at FC1 (blue circle). Resulting predicted field potentials and extent of current flow within the brain is shown on axial, coronal, and sagittal brains.

A 4 × 1 HD-tDCS adapter connected to a 1 × 1 Clinical Trial system (Soterix Medical Inc., New York, NY, USA) was used to deliver active (anodal) and sham stimulation. Optimal electrode configuration for stimulation of left SMA was determined using HD-Explore^TM^ software (Soterix Medical Inc.,) modeling software that provides the predicted electrical current flow within the brain during stimulation (Figure 2). Ag/AgCl sintered ring electrodes were positioned within electrode holders filled with electrolyte gel in a head cap, which uses the International 10–20 System for EEG to label electrode positions. We centered position Cz on the head at the halfway point between the nasion and the inion longitudinally and left to right pre-auricular areas laterally. Stimulation condition delivery was controlled by a six-digit numeric code input into the stimulation device that either delivered active or sham stimulation. Further, the experimenter who entered the code into the device was blind with regard to which type of stimulation it initiated. During active stimulation, the current was ramped up over 30 s, maintained at 1.5 mA for the rest of the session, and automatically ramped down at the end of the 20-min session. The stimulation intensity in the present study is well within the typical safe and tolerable range used in human studies (Bikson et al., 2016; Woods et al., 2016). For sham stimulation, the current was automatically ramped up and down over the first 30 s to prevent actual stimulation of the target region. The current also was ramped up and back down 30 s prior to the end of the 20-min session, consistent with the literature, to assist with participant blinding (but see Richardson et al., 2014; Garnett and den Ouden, 2015; Woods et al., 2016; Fonteneau et al., 2019; for other methods of sham delivery). During stimulation participants read aloud while pacing each syllable to the beat of a metronome. The metronome speed was set at the maximum comfortable rate for each participant.

### fMRI Procedure

The protocol for each fMRI session (i.e., before and after stimulation in both visits) was the same. Participants read aloud in two conditions: choral reading and solo reading. During choral reading, participants synchronized their reading with an audio recording (*via* earphones) of the same passage presented on the screen. During solo reading, participants read at their natural speech rate and backwards speech was presented in the earphones. This was designed to minimize differences in background auditory feedback between the conditions. In each of four 7-min functional runs, four 30-s blocks of solo reading and four blocks of choral reading were presented in alternating order. Scans were acquired using a 3T GE MRI scanner (MR 750). A standard echoplanar (EPI) pulse sequence was used, with the following parameters: recovery time (TR) = 2 s; voxel size = 3.4 × 3.4 × 4 mm; 37 interleaved sagittal slices; acceleration factor = 2. In addition, high-resolution structural images were acquired at the beginning of each scanning session using spoiled gradient-recalled acquisition in steady-state (SPGR) imaging (flip angle = 15°, FOV = 256 mm, 1 mm slice thickness).

### fMRI Data Analysis

SPM12^1^ was used for fMRI data preprocessing and statistical analysis unless specified otherwise. For each participant, functional images were corrected for differences in slice acquisition timings. Anatomical scans and functional volumes were co-registered to the first volume of the first scan using rigid body rotation. Functional scans were concatenated and de-noised using a strategy detailed in our previous publication (Xu et al., 2014). This de-noising strategy is based on spatial Independent Component Analysis (sICA), a signal un-mixing algorithm, and has been demonstrated to be able to remove fMRI artifacts associated with continuous speech production (AbdulSabur et al., 2014). De-noised functional scans were normalized to MNI space and spatially smoothed with a 6 mm FWHM kernel. Each participant’s preprocessed data were analyzed using general linear model (GLM) implemented in SPM12. Speaking conditions (choral and solo) at each stimulation condition (active and sham) were modeled with separate regressors. Individual beta estimates of each condition and participant’s SSI score as a covariate were entered into group-level analysis. Pairwise *t*-tests were used to compare between conditions. Family-wise errors were controlled using voxel-wise height threshold *p* < 0.001 and cluster-size threshold *k* > 12 voxels, which corresponded to a corrected *p* < 0.05. The cluster-size threshold was determined by AFNI 3dClustSim (version 17.2.13) with non-Gaussian auto-correlation function (-acf option; Cox et al., 2017).

## Results

### Effects of Anodal Stimulation on Brain Activity During Continuous Speaking

The first goal of this study was to examine the effects of HD-tDCS on brain activity during continuous speech. We contrasted the brain activity after active stimulation with that after sham stimulation, resulting in the (Active-Sham) contrast. For the purposes of this study, and due to lack of differential neural activity in the two speaking conditions, we report results with choral speech and solo speech combined: [active (choral+solo)] − [sham (choral+solo)]. Results for this analysis did not reveal any clusters that reached the pre-set significance threshold.

### Modulatory Effects of Stuttering Severity

**Figure 3:**
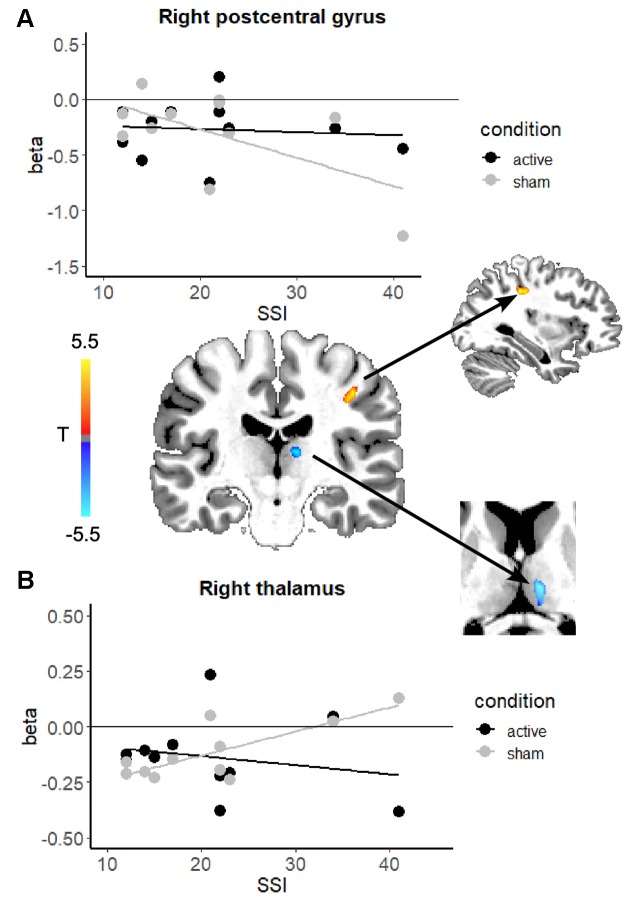
Scatterplots showing the relationship between brain activation and stuttering severity (SSI-4 scores) after active stimulation (black) and after sham stimulation (gray) the right postcentral gyrus **(A)** and the right thalamus **(B)**. In the center, the *change* in the relationship between stuttering severity and brain activity in each region is overlaid on an example individual anatomical image. In both regions, active stimulation attenuated the relationship between severity and brain activation. Color blobs represent the overall direction of the change in correlation: in the right postcentral gyrus the relationship became *less negative*, while in the right thalamus the relationship became *less positive*.

The second goal of the study was to determine if stuttering severity modulated the effects of stimulation on brain activity during speech. Given the range of severity in our initial 14 subjects, we excluded three subjects whose SSI scores were 10 or below (note: we also ran the analysis from “Effects of Anodal Stimulation on Brain Activity During Continuous Speaking” section excluding these same subjects, which did not change the results of that analysis). In this analysis of 11 subjects, the contrast involved subtracting the *severity-modulated activation* during sham from that during active. This leaves us with the *change* in relationship between severity and neural activation due to active stimulation. In other words, these are the regions that exhibited a differential modulatory effect of severity in speech-related brain regions. Table 1 and Figure 3 shows modulatory effects of stuttering severity on brain activity during speaking (natural and fluent conditions combined). Two clusters showed a differential modulatory effect of stuttering severity after active vs. sham stimulation for the speaking conditions combined: the right posterior central gyrus and the right thalamus.

**Table 1:** Regions that exhibited a significant modulatory effect of SSI-4 scores on brain activity.

Hemisphere	Peak region	# voxels	*x*	*y*	*z*	*t*
R	Postcentral gyrus	37	33	−33	39	5.49
R	Thalamus	15	12	−21	9	−5.10

### Effects of Stimulation on Speech Fluency

During the reading portion, speech fluency significantly increased (%SLD decreased) following both active and sham stimulation. The %SLD before active stimulation (M = 7.52) decreased after stimulation (M = 3.97;* p* = 0.022). The %SLD before sham stimulation (M = 6.83) decreased after sham stimulation (M = 3.97; *p* = 0.027). Active stimulation had a greater effect on %SLD, resulting in a decrease of 3.55% compared to 2.86% after sham stimulation. There were no significant effects of stimulation or training alone (sham) on speech fluency during the conversation portion of the speech sample.

## Discussion

As a preliminary window into the possible therapeutic use of brain stimulation to augment the effects of speech therapy, we investigated, for the first time, the effects of focal neuromodulation using HD-tDCS on speech fluency and brain activity during continuous speech in AWS. We expected that compared to sham stimulation, active stimulation would increase activity of areas functionally connected to SMA, such as BGTC network areas, in turn enhancing speech timing and thus fluency. Namely, that traditionally underactive regions (e.g., left IFG, basal ganglia) would show enhanced activity after anodal stimulation. Results from the main effect of active-sham stimulation analysis did not support our hypotheses. Any changes in brain activity or functional connectivity as a result of active stimulation of left SMA did not differ significantly from that observed in the sham condition.

There are at least three potential explanations for the lack of significant effects in this first analysis. First, due to the relatively small sample size, it is possible that we did not have enough statistical power to detect differences. However, even when examining the data at subthreshold there was very little difference between active and sham stimulation. Second, the SMA may not have been an ideal choice as a target for stimulation. Although SMA plays a critical role within the BGTC network in initiating complex motor sequences and has major connections with the putamen as well as the IFG, it is possible that the timing (as opposed to simply increased activity levels) of SMA activity in relation to movement onset is more important in augmenting fluent speech motor control. The current experimental design did not allow for such an examination. Third, there are considerable individual differences among PWS including severity levels, as well as possible SMA connectivity. In the current study, we used a modeling software that estimated spread of electric fields as a result of stimulating a specific target area, which was based on a single template brain that was applied to individual AWS to help localize and apply the electrodes to their scalp. In future studies, applying a subject-specific stimulation target localization based on the individual subjects’ brain structural MRI data might help mitigate these issues.

One variable that can differ considerably across speakers is stuttering severity. To account for the effects of stuttering severity, we examined the modulatory effects of stuttering severity on brain activity in a second analysis. Interestingly, we found two clusters in the right hemisphere that were differentially affected by active vs. sham stimulation in relation to severity. Active stimulation seems to deliver a stronger effect to severe AWS, changing the activation levels in regions that were abnormally heightened in severe AWS to be more similar to that in the mild AWS. Taken together, the two regions in which active stimulation modulated the relationship between activity and stuttering severity are part of the BGTC network that supports speech processing (Kotz and Schwartze, 2010). Within the BGTC network, the thalamus is suggested to play a critical role in interfacing the cerebellum with cortical motor areas (Kotz et al., 2016).

In the right postcentral gyrus, the negative correlation between stuttering severity and brain activity (gray line) was no longer present after active stimulation (black line). The right postcentral gyrus exhibited greater activity for more severe AWS at baseline (before active stimulation; Figure 2, gray line). This relationship, however, was no longer present after active stimulation. The results of the severity modulation somewhat support our hypotheses. These areas are known to mediate somatosensory processing, timing, and motor control. In a recent meta-analysis (Neef et al., 2015), the right postcentral gyrus was more active in PWS than fluent speakers (group effects, not severity). We found that although there was no main effect of stimulation in this region, active stimulation attenuated a negative relationship between activity in the right postcentral gyrus and stuttering severity.

In the right thalamus, we found that a positive relation between stuttering severity and activation in the right thalamus is no longer present after active stimulation. This finding may be related to findings from Giraud et al. (2008), who reported that stuttering severity was positively correlated with BG activity before, but not after therapy that focused on fluency-inducing techniques. Similar results have been found for the putamen (Neumann et al., 2003). In another study, Neef et al. (2018) used fMRI-based tractography to find that stuttering severity was positively correlated with connection strength between the right anterior thalamic radiation with seed placement in the right frontal pole. These results suggest that hyperactivity and connectivity involving the thalamus in more severe AWS could be alleviated with tDCS.

Finally, both active and sham stimulation significantly decreased the amount of stuttering during the reading portion of the speech sample, but not during conversation. This result is in agreement with the results of Chesters et al. (2018) who found that the effect of their tDCS treatment on reading (but not conversation) lasted up to 6 weeks. Although in the present study active stimulation led to a greater decrease (3.5%) in stuttering compared to sham stimulation (2.86%), this difference was not significant. This is not unexpected given that participants only received one session of active stimulation, which could be argued to be insufficient for effecting change in a complex motor movement like speech.

In conclusion, we report preliminary findings from the first HD-tDCS study of AWS. We found that anodal stimulation to the left SMA modulated the relationship between brain activity and stuttering severity in key regions of the right thalamocortical network. This is an important first step in paving the way for future use of HD-tDCS in the treatment of stuttering, which may have greater efficacy for treatment of severe stuttering cases. However, these results should be interpreted with caution due to some limitations, including the small sample size and wide range of stuttering severity represented in the current subject sample. Stuttering severity was measured using the SSI, and like all measures of behavioral attributes of stuttering, the score is influenced by many factors, including fatigue, task demands (e.g., reading vs. conversation), and treatment history, among others. All participants in this study reported receiving at least some formal stuttering therapy during their school years, with the exception of one who sought treatment in college, but none within 1 year of study participation. Because each person was his or her own control, differences in the individual before and after stimulation, accordingly, confounds (e.g., treatment history and severity) across individuals were minimized.

It is also possible that the use of metronome-paced reading during stimulation but choral reading during fMRI may have affected the results. During stimulation, we sought to use a condition that maximized chances of enhancing fluency in speakers who stutter, and metronome-paced speech is such a condition, even compared to choral speech (Toyomura et al., 2011). We paired stimulation with this condition to heighten the chances of augmenting the effects of the fluency inducing condition, and thus increase chances of finding differences between active and sham stimulation. There were also practical reasons behind using choral reading during fMRI. The auditory beats of the metronome could interact with the rhythmic beats and sounds of the scanner, and lead to additional auditory stimulation. Instead, we chose to use choral speech, which elicits similar neural activity (albeit weaker) than metronome-paced speech (Toyomura et al., 2011). Choral reading is also somewhat more naturalistic because it involves human voice rather than beats. Still, the lack of significant findings could be related to the use of different tasks, i.e., the effect could not transfer from one task to another.

Finally, this study involved a single active tDCS session, which likely provided insufficient intensity to precipitate significant changes in brain activity and speech fluency in all our participants. Chesters et al. (2018) have preliminary evidence showing that intensive treatment that involves delivering tDCS on five consecutive days was needed to effect behavioral change. This is consistent with other studies that have used similar multiple-day treatment designs that led to significant and prolonged motor enhancement (e.g., Reis et al., 2009). Future studies with larger sample sizes and more intensive delivery of stimulation are needed to further our understanding of effects of brain stimulation on brain activity and speech fluency. Other stimulation target regions should also be explored, guided by previous neuroimaging findings in stuttering.

## Data Availability Statement

The raw data supporting the conclusions of this manuscript will be made available by the authors to any qualified researcher.

## Ethics Statement

The studies involving human participants were reviewed and approved by Institutional Review Boards of the University of Michigan Medical School (IRBMED) which oversees human subjects research conducted at the Medical School and Michigan Medicine. The participants provided their written informed consent to participate in this study.

## Author Contributions

EG, HC, AC, and S-EC contributed to the conception and design of the study. EG and AC recruited participants and collected the data. EG and HC performed data analysis. EG wrote the first draft of the manuscript. All authors contributed to manuscript revisions and read and approved the submitted version.

## Conflict of Interest

The authors declare that the research was conducted in the absence of any commercial or financial relationships that could be construed as a potential conflict of interest.

## Footnotes

^1^https://www.fil.ion.ucl.ac.uk/spm/software/spm12/
